# The protective effect of *Phellinus linteus* decoction on podocyte injury in the kidney of FSGS rats

**DOI:** 10.1186/s12906-019-2705-3

**Published:** 2019-10-21

**Authors:** Feng Wan, Ru-chun Yang, Yan-peng Shi, Yue-Wen Tang, Xuan-li Tang, Xiao-ling Zhu, You-gui Li, Yong-jun Wang

**Affiliations:** 1 Department of Nephrology (Key laboratory of Zhejiang province, management of kidney disease), Guang Xing Hospital Affiliated to Zhejiang Chinese Medical University, Hangzhou, 310007 Tiyuchang Road 453, People’s Republic of China; 2 Sericultural Research Institute, Zhejiang Academy of Agricultural Science, Hangzhou, China

**Keywords:** *Phellinus linteus* decoction, Protective effect, Podocyte injury, FSGS, Rat

## Abstract

**Background:**

This study aimed to investigate the effect of the *Phellinus linteus* (Mesima) decoction on podocyte injury in a rat model of focal and segmental glomerulosclerosis (FSGS) and evaluate the potential mechanisms.

**Methods:**

FSGS resembling primary FSGS in humans was established in rats by uninephrectomy and the repeated injection of doxorubicin. The FSGS rats were randomly divided into the model group, low-dose group of *P. linteus* decoction (PLD-LD), medium-dose group of *P. linteus* decoction (PLD-MD), and high-dose group of *P. linteus* decoction (PLD-HD). Blood and urine analysis were performed after 12 weeks and the molecular indicators of renal function and the renal pathological changes were examined.

**Results:**

FSGS developed within 12 weeks in the test group and showed progressive proteinuria and segmental glomerular scarring. Urinary protein, serum creatinine, urea nitrogen, triglycerides and cholesterol were significantly reduced following the 12-week intervention with *P.linteus* decoction, especially in the PLD-LD group. Renal nephrin and podocin were markedly increased. Moreover, the pathological damage in the renal tissue was alleviated by the PLD-LD intervention.

**Conclusion:**

The *P. linteus* decoction alleviated the podocyte injury in the FSGS rat model, thus minimizing the progression of glomerular sclerosis and improving renal function.

## Background

Focal and segmental glomerulosclerosis (FSGS) represents a frequently occuring glomerular kidney disease [1]. It is usually delineated as a clinical-pathologic syndrome manifesting proteinuria, and focal and segmental glomerular sclerosis with foot process effacement [2]. The main clinical manifestation of FSGS is proteinuria. Currently, the first-line of treatment in idiopathic FSGS with nephritic syndrome is a prolonged course of corticosteroids [3]. Unfortunately, the occurrence of steroid resistance or steroid dependence is commonly reported. FSGS may still result in end-stage renal failure despite intensified immunosuppressive therapy. Thus, it remains an enormous challenge to find novel and effective treatments for FSGS.

Traditional Chinese medicines have been considered as effective treatments for a variety of different physical conditions, including renal diseases [4]. *Phellinus linteus* (Mesima), a kind of mushroom that grows mainly on wild mulberry tree trunks, is used extensively as a traditional medicine in China, Korea, Japan, and other Asian countries for the treatment of different diseases [5]. The main biological functions of *P. linteus* include anti-cancer, antioxidant, anti-inflammatory, hypoglycaemic and anti-fibrotic [6–10]. Considering its remarkable anti-cancer effects, *P. linteus* has become a research hotspot at home and abroad. However, its application in the treatment of kidney diseases at home and abroad is scarce.

As is known, many pathological factors played important roles during the occurrence and progression of kidney disease, including oxidative stress, inflammatory reaction, immune disorder, and disturbances of glucose and lipid metabolism. These coincide with the multifaceted pharmacological effects of *P. linteus* mentioned above, which make us postulate that *P. linteus* may have protective effect on the kidney. Expectedly, in our previous study, we found different extracts from *P. linteus* can inhibit TGF-β1-induced epithelial-mesenchymal transition in renal tubular epithelial cells (NRK-52E) [11]. Additionally, it is worth noting that a recent literature showed that polysaccharides from *P. linteus* can reduce renal interstitial fibrosis in diabetic nephropathy mice [12]. These indicated *P. linteus* had great potential in preventing and protecting kidney disease. However, further validation is still needed.

In this current study, we established a FSGS rat model through uninephrectomy and repeated doxorubicin administration. Next, we evaluated the potential therapeutic effects of *P. linteus* on the FSGS rat. We assessed the urinary protein levels, kidney function, expression of podocyte slit diaphragm proteins (nephrin and podocin), and the pathomorphology of the FSGS kidney tissues in the different treatment groups of rats. Our results elucidated the potential therapeutic value of *P. linteus* in the treatment of FSGS.

## Methods

### Preparation of *P. linteus* decoction

The powder of *P. linteus* (Mesima) was prepared and provided by professor You-gui Li, Zhejiang Academy of Agricultural Science. The *P. li*nteus used in this study was authenticated by the Institute of Microbiology of Chinese Academy of Sciences. The specimen of *P. linteus* has been kept at the herbarium of Zhejiang Academy of Agricultural Science.

According to the ancient books (Sheng ji zong lu and Pu ji Fang), the recommended clinical dosage of P. linteus is about 10 g/d for adults. The appropriate dosage for each rat is calculated on the basis of body surface area [13]. In our experiment, in combination with some preliminary experiments, we finally chose the dosage of PLD-LD, PLD-MD, and PLD-HD is 4 g /d, 8 g/d, and 16 g/d, respectively. The decoction was prepared as follows: the powder was initially soaked in distilled water for half an hour, and then decocted for 30 min two times, in accordance with conventional method. Finally, the decoctions were combined and filtered using a double-layer gauze, and concentrated to the required volume for spare.

### Experimental animals

Clean-grade male Sprague Dawley rats (*n* = 30) weighing 160-180 g were purchased from the Zhejiang Institute of Traditional Chinese Medicine (animal qualification certificate number: SCXK [shanghai] 2013–0016). The rats were housed under standard conditions and the experiments were performed in accordance with the local guidelines of animal experiment center, for the care of laboratory animals. All animal experiments were approved by the ethics committee for research on laboratory animal use of the Zhejiang Institute of Traditional Chinese Medicine (Tianmushan Road No.132).

### Study of *P. linteus* in FSGS rats

The rats were allowed to acclimatize for a week prior to stating the experiments, and then weighed and numbered according to their body weight (from light to heavy). At first, six rats were randomly selected as the control group by the random number table. The remaining 24 rats were used to establish the FSGS model (*n* = 6 rats/group). Based on the proteinuria difference between the control and model group in our preliminary experimental results, the total rat numbers in our study were determined. Thereafter, the FSGS rat model was established as previously described [4, 13, 14]. Briefly, the rats were firstly subjected to uninephrectomy (left side) on day 1, followed by the administration of doxorubicin through caudal vein, 5 mg/kg (on day 7) and 3 mg/kg (on day 28), respectively. For the control rats, they were correspondingly injected equivalent saline on day 7 and day 28 following the sham operation.

The FSGS rats were allocated randomly to four groups: model group, PLD-LD, PLD-MD, and PLD-HD (*n* = 6/each group). The intervention with different concentrations of *P. linteus* decoction by gavage was initiated on day 2. After administration for eight consecutive weeks, the serum and whole right kidneys were harvested for biochemical, histological, and molecular analyses, and followed by the euthanisation of the animals by dislocation of the cervical spine. Urine samples were collected for 24 h by using the metabolic cages. The urinary protein level in the rats was quantified by a biochemical analyser (HITACHI 7180).

### Histological analysis

A portion of kidney was fixed with 4% paraformaldehyde and embedded in paraffin. 3 μm-thick sections were cut and stained with haematoxylin and eosin (H&E) and Masson’s trichrome stain for examination of kidney histology. The degree of sclerosis in each glomerulus was subjectively graded on a scale of 0 to 4 as described previously [13]. The glomerular sclerosis index (GSI) was calculated by using the following formula as previously reported:

$\mathrm{GSI}=\frac{\left(1\times \mathrm{N}1\right)+\left(2\times \mathrm{N}2\right)+\left(3\times \mathrm{N}3\right)+\left(4\times \mathrm{N}4\right)}{\mathrm{N}0+\mathrm{N}1+\mathrm{N}2+\mathrm{N}3+\mathrm{N}4}$, where N is the number of glomeruli at each grade of sclerosis.

### Transmission electron microscopy (TEM)

After fixation in 2.5% glutaraldehyde overnight, the kidney tissue (~ 1 mm^3^ in size) was rinsed in 0.1 M PBS thrice. Then the specimens were post-fixed with 1% osmium tetroxide for 1 h, dehydrated in graded series of acetone and embedded in graded Epon 812. Ultrathin sections (80–100 nm) were cut and stained with uranyl acetate (2%) and lead citrate [15], and observed with a JEM-1400 transmission electron microscope (JEOL, Japan).

### Real-time quantitative PCR (qRT-PCR)

Total RNA was isolated from kidneys of individual rats using the TRIzol reagent (Invitrogen, USA) and then cDNA was synthesized using a Primescript™ RT reagent kit (TaKaRa, Japan) according to the manufacturer’s instructions. Thereafter, expression levels of nephrin, podocin, and GAPDH were quantified via real-time PCR using Applied Biosystems® 7500 Fast real-time PCR system (Thermo Fisher Scientific, USA). The sequences of primers used are shown in Table 1. Relative mRNA expression levels were normalized to those of GAPDH. Each PCR experiment was performed in triplicate and repeated independently at least thrice.

**Table 1:** Primers used for gene expression analyses. F, forward primer; R, reverse primer

Primer Name	Sequence (5′ to 3′)	Application
Rat-nephrin-F	GTTGGTGGTCTTCTGCTGCTCTC	Real-time RT-PCR
Rat-nephrin-R	CTTCTGCTGTGCTAACCGTGGAG	Real-time RT-PCR
Rat-podocin-F	CCAGCAGCCACGGTAGTGAATG	Real-time RT-PCR
Rat-podocin-R	CCTCTGGTCGCTCGCTCTCC	Real-time RT-PCR
Rat-GAPDH-F	ACCACAGTCCATGCCATCAC	Real-time RT-PCR
Rat-GAPDH-R	TCCACCACCCTGTTGCTGTA	Real-time RT-PCR

### Western blotting

The rat kidney tissues were incubated with RIPA lysis buffer [20 mM Tris (pH 7.5), 150 mM NaCl, 1% Triton X-100, 1% sodium deoxycholate, 0.1% SDS and 1 mM EDTA] containing a proteinase inhibitor cocktail (Beyotime) at 4 °C for 30 min. Cell lysates were centrifuged at 10,000 g for 10 min at 4 °C. After quantification using the BCA assay, the protein extracts (80 μg) were separated by 10% SDS-PAGE gels under reducing conditions. After the proteins were transferred onto a PVDF membrane, 5% skimmed milk was used as the blocking agent, and then the membranes were next incubated with the primary antibodies. Antibodies used were as follows: anti-nephrin (Abcam, ab58968); anti-podocin (Abcam, ab93650); and anti-GAPDH (ProteinTech, 60,004–1-Ig). After hybridization, the blots were washed and incubated with infrared labelled anti-rabbit/mouse IgG Ab (1:15000). Finally, the signal was detected using an Odyssey CLx image system (LI-COR).

### Statistical analysis

Data are presented as means ± SD values. The data shown were analysed for significance via Student’s t-test or One-Way ANOVA using SPSS20.0 software (IBM Corporation, Armonk, NY, USA). A *p*-value less than 0.05 or 0.01 was considered statistically significance.

## Results

### Effect of *P. linteus* decoction on urinary protein excretion in FSGS rats

**Fig. 1:**
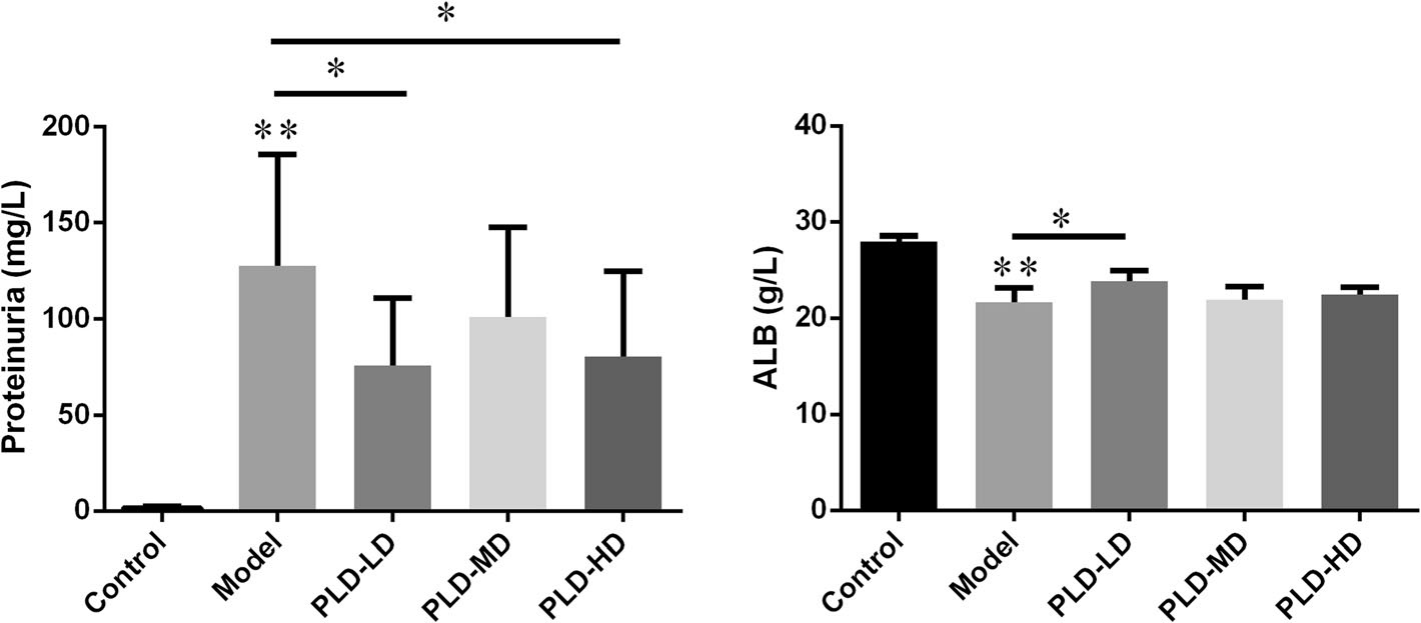
Evaluation of proteinuria and serum ALB concentration. The 24 h urine and serum ALB concentrations were collected from experimental rats. Data (*n* = 6) are presented as the mean ± SD. ^*^p < 0.05; ^**^p < 0.01

To establish a model for rat FSGS, we subjected rats to uninephrectomy followed by repeated injection of doxorubicin. The urinary protein levels for FSGS rats were significantly higher than that of control rats (*p* < 0.01, Fig. 1a), indicating that the FSGS model had been successfully established. No rat mortalities were recorded during the course of the whole experiment. Besides, the serum albumin (ALB) level in the FSGS model decreased significantly compared with the control group (*p* < 0.01, Fig. 1b). After the intervention with the *P. linteus* decoction, the 24 h urinary protein levels in PLD-LD and PLD-HD groups were much lower than that in the model group (Fig. 1a). Meanwhile, the ALB in the PLD-LD group showed obvious improvement (Fig. 1b). No significant differences in the ALB levels between the model group and PLD-MD and PLD-HD group were observed. Collectively, these results indicate that the PLD-LD can attenuate urinary protein excretion and improve the serum ALB levels in the FSGS rat.

### Effect of *P. linteus* decoction on renal function in FSGS rats

**Fig. 2:**
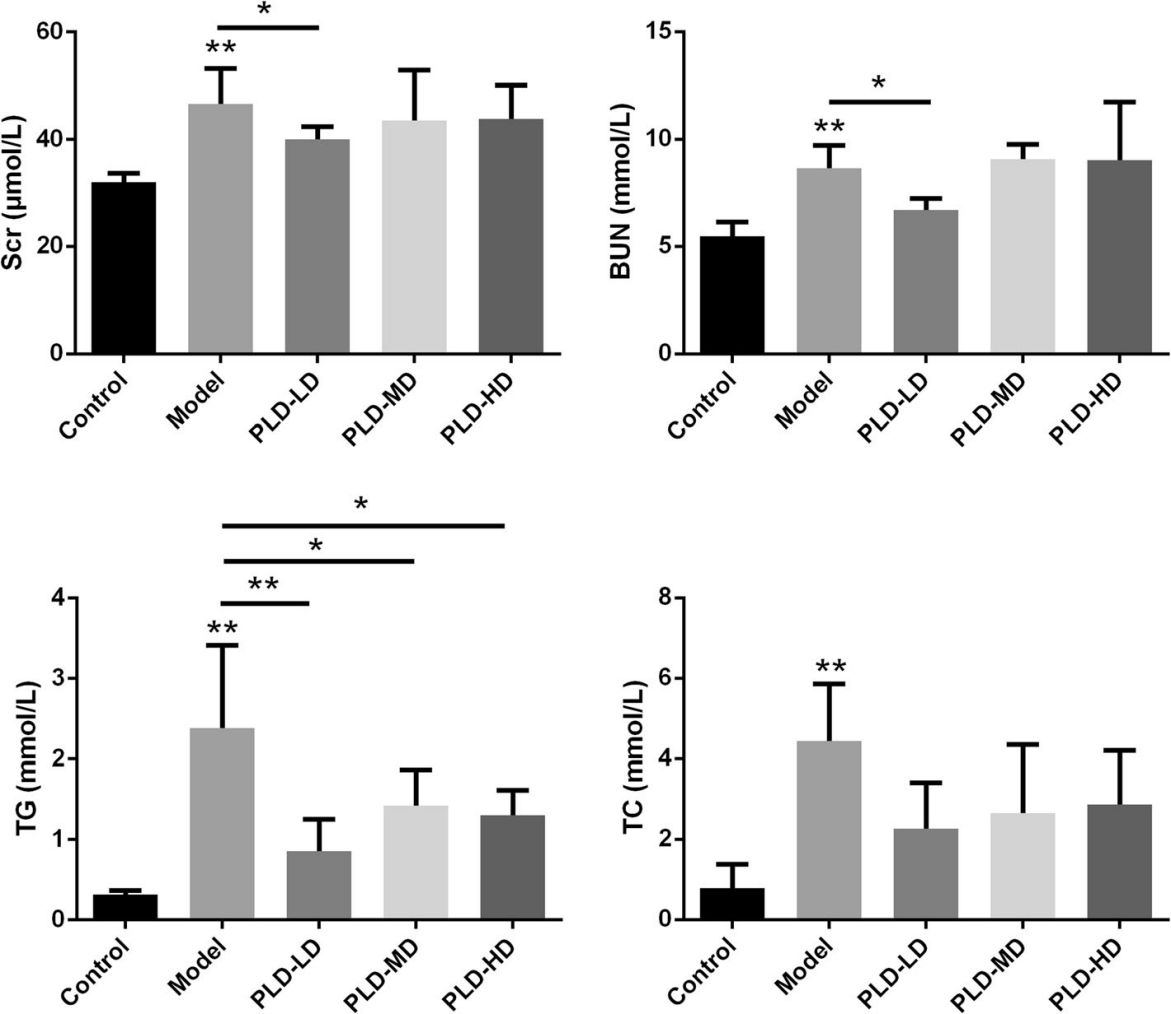
Evaluation of renal function. The levels of Scr (μmol/L), BUN (mmol/L), TG (mmol/L), and TC (mmol/L) in serum from different groups of rats were determined by a chemical analszer (HITACHI 7180). Data (*n* = 6) are presented as the mean ± SD. ^*^p < 0.05; ^**^p < 0.01

To further evaluate the effects of the *P. linteus* decoction on kidney function, the serum markers in different groups were measured. Serum creatinine (Scr), urea nitrogen (BUN), triglycerides (TG), and cholesterol (TC) were significantly increased in the FSGS group compared with the control group (Fig. 2). Additionally, the Scr, BUN, and TG were significantly reduced in the PLD-LD group compared with the model group. However, there were no significant differences in TC levels between the model group and the PLD-LD, PLD-MD and the PLD-HD groups (Fig. 2). These results are basically in line with the data from urinary protein, providing further evidence that PLD-LD could ameliorate kidney damage.

### Effect of *P. linteus* decoction on glomerular pathomorphology in FSGS rats

**Fig. 3:**
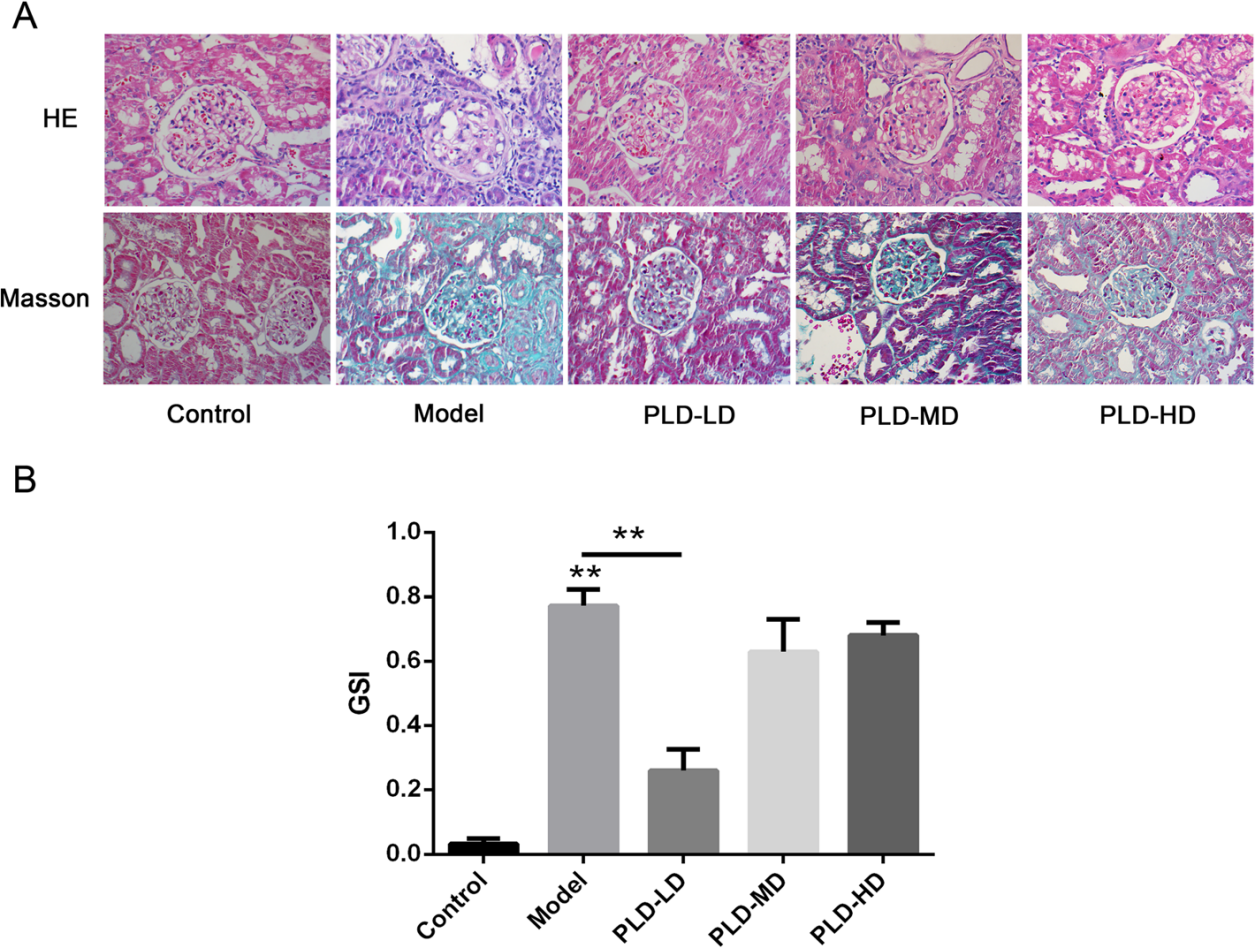
Analysis of renal pathology by light microscopy. **a** Representative micrographs from each group are shown. The upper panel represents H & E staining and the lower panel represents the Masson’s staining (original magnification × 200). **b** The GSI of each group. Data are presented as the mean ± SD. ^*^p < 0.05; ^**^p < 0.01

The glomerular pathomorphology was further examined by histological analysis. H&E and Masson’s staining demonstrated that there was obvious focal glomerular sclerosis, interstitial lesions, and inflammatory cell infiltration in the model rats, whereas the control rats had no pathological changes (Fig. 3a). Furthermore, the GSI in the model group was much higher than that in the control group (Fig. 3b). After the *P. linteus* decoction intervention, such pathological damages were much improved compared with that in the model group, especially the PLD-LD group (Fig. 3a). Similarly, the GSI in the PLD-LD group decreased significantly compared to that in the model group (Fig. 3b).

**Fig. 4:**
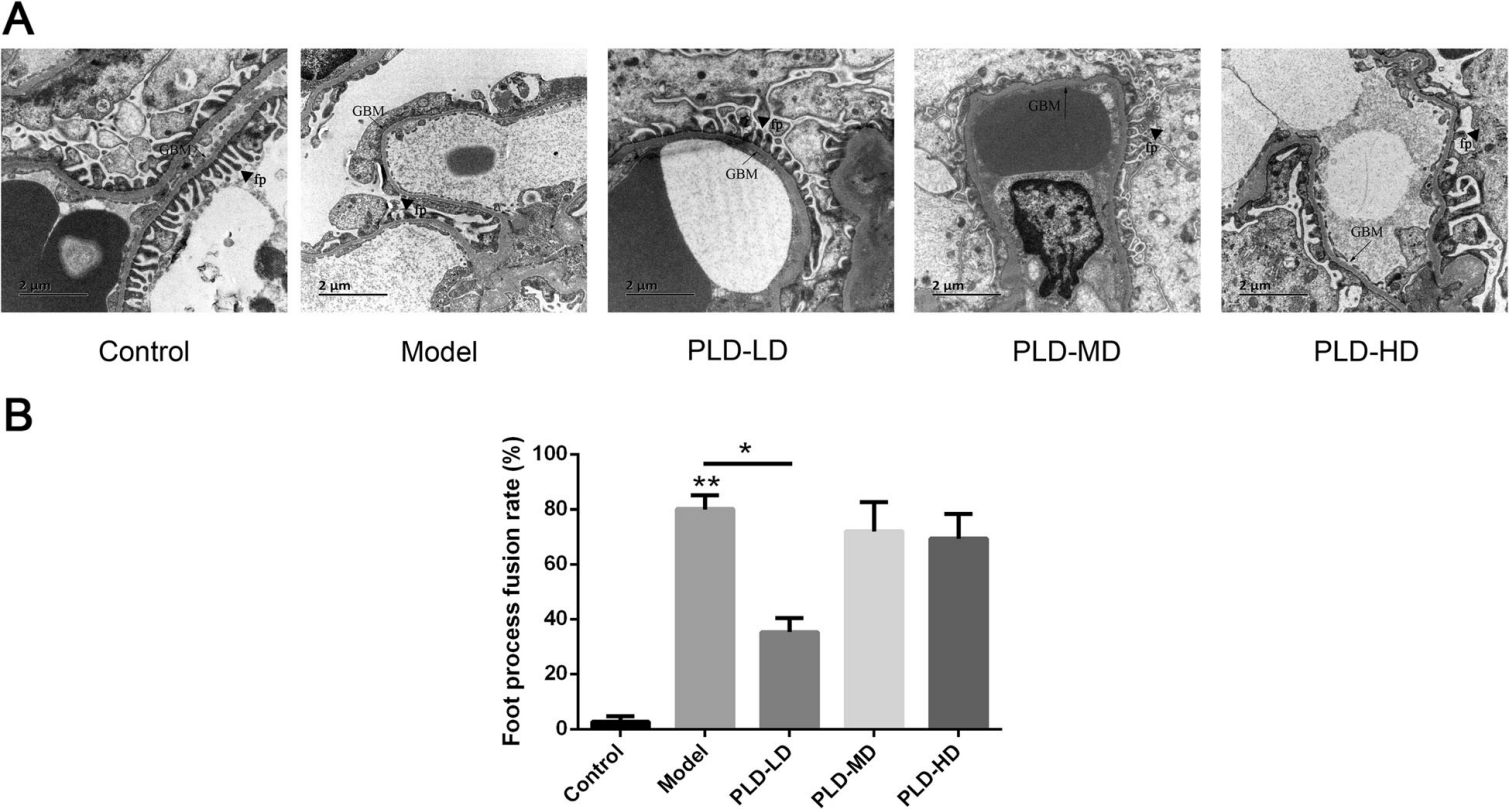
Analysis of renal pathology by TEM. **a** Representative micrographs in each group are shown. GBM, glomerular basement membrane (black arrow); fp, podocyte foot process (black triangle). Scale bar 2 μm (bottom left). Original magnification × 10,000, n = 6 animals in each group. **b** The foot fusion rate of each group. Data are presented as the mean ± SD. ^*^p < 0.05; ^**^p < 0.01

Next, TEM was used to observe the degree of damage in the podocyte foot process. The results showed clear processes were observed on the surface of the glomerular epithelial cells in the control group (Fig. 4a). However, the foot processes diffusely effaced, became flat and fused and even disappeared in the model group. Compared with the control group, the foot process rate in the model group increased from 2.67 ± 1.00 to 80% ± 6.12% (Fig. 4b, *p* < 0.01). In the PLD-LD group, the lesions were significantly alleviated and foot fusion rate (35.33% ± 5.03%) reduced compared with the model group. However, the lesions in PLD-MD and PLD-HD groups were much more serious than that in the PLD-LD group (Fig. 4a). Taken together, these results indicated that the PLD-LD treatment can alleviate the renal pathological damage.

### Effect of *P. linteus* decoction on renal nephrin and podocin expression in FSGS rats

**Fig. 5:**
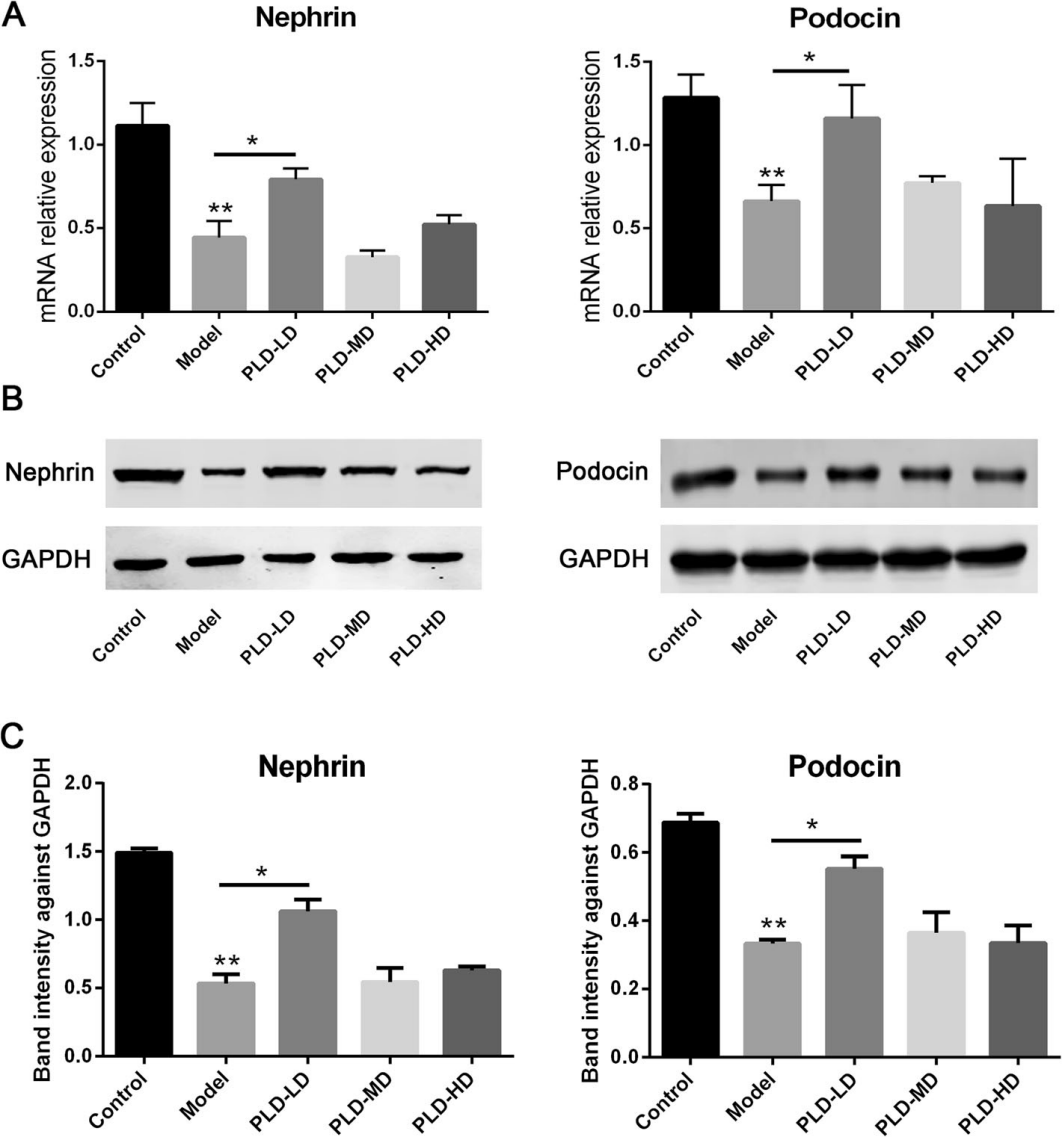
Analysis of the expression of nephrin and podocin. **a** The mRNA expressions of nephrin and podocin in the rat kidney tissues were determined by qRT-PCR. The relative gene expression was normalized to that of GAPDH. Quantitative data are shown as the mean ± SD. ^*^p < 0.05; ^**^p < 0.01. **b** Representative western blot of nephrin and podocin in the rat kidney tissues. **c** Relative protein level was calculated by band intensity against GAPDH, respectively

To further assess the effect of the *P. linteus* decoction on FSGS progression, the podocyte slit diaphragm proteins, nephrin and podocin were assessed. RT-PCR results showed the expression levels of nephrin and podocin were markedly decreased in the model group (Fig. 5a). In the PLD-LD intervention group, the expression of nephrin and podocin were increased significantly compared with that in the model group. However, there was no significant difference between the model and PLD-MD and PLD-HD groups (Fig. 5a). Similar results were observed by western blotting (Fig. 5b and c). These results suggested that PLD-LD can protect the podocyte slit diaphragm proteins nephrin and podocin.

## Discussion

FSGS represents a major cause of the nephritic syndrome. It is the most common primary glomerular disorder causing ESRD. Hormones and immunosuppressants are the most extensively used agents in the treatment of FSGS. However, this therapeutic strategy is not considered ideal due to the occurrence of unpleasant side effects and steroid-resistance. Steroid-resistant patients with FSGS are of great concern to nephrologists as these patients are at significant risk for ongoing progression of ESRD. Therefore, there is an urgent need to seek a safer and more effective method to relieve FSGS progression.

*P. linteus*, a well-established medicinal mushroom, is also known as “forest gold”. It has been used in Asian countries for centuries to prevent or treat diseases as diverse as haemorrhage, rheumatoid arthritis, gastroenteric dysfunction, diarrhoea, and cancers [16]. Recent studies have demonstrated that *P. linteus* has anti-hepatic fibrosis effect [10]. Considering its extensive biological role, *P. linteus* has become a hot research topic in the medical research, and has attracted much attention from scholars at home and abroad. Although the research on *P. linteus* includes many fields, until now, studies on its protective roles in chronic kidney disease have not been reported.

Podocyte, an important component of the glomerular filtration membrane, plays an important role in the development of FSGS [17]. Podocyte injury is a common feature of many glomerular diseases, which can lead to foot process fusion and cell detachment from the glomerular base membrane, thus resulting in proteinuria and glomerulosclerosis [18]. Nephrin is the main and the most abundant protein of the podocyte slit diaphragm (SD) [19]. Podocin is a membrane protein located exclusively in the SD region, with two intracellular domains that interact with nephrin. Changes in nephrin and podocin structures can lead to intense proteinuria [20, 21]. In our study, PLD-LD partially reduced the fusion and effacement of the foot process, and increased nephrin and podocin expression, suggesting that podocyte injury is closely associated with the incidence of glomerular sclerosis in our model, and PLD-LD may attenuate the progression of FSGS through protection afforded to podocyte injury.

A potential limitation of our study was the failure to demonstrate a dose-dependent effect among PLD-LD, PLD-MD and PLD-HD groups. One possible explanation could be that the dose range we used was too high for rats. Therefore, further studies including much smaller doses are needed to ascertain the exact dose range and effect of *P. linteus* in FSGS rats.

In summary, this study revealed for the first time that *P. linteus* decoction can protect the podocyte injury in the kidney of FSGS rats. It is anticipated that this study can provide a theoretical basis for the application of *P. linteus* in the treatment of FSGS.

## Conclusions

In conclusion, FSGS resembling primary FSGS in humans was established in rats by uninephrectomy and the repeated injection of doxorubicin. Then the effect of *P.linteus* decoction on FSGS rats was evaluated in this paper. After 12-week intervention with *P.linteus* decoction, urinary protein, Scr, BUN, TG and TC were significantly reduced in our experiment, especially in the PLD-LD group. The expression of renal nephrin and podocin were increased significantly. Moreover, the pathological damage in the renal tissue was also alleviated by the PLD-LD intervention. Taken together, the *P. linteus* decoction can alleviate the podocyte injury in the FSGS rat model, thus minimizing the progression of glomerular sclerosis and improving renal function.

## Data Availability

The datasets used and analyzed during the current study are available from the corresponding author on reasonable request.

## Abbreviations

FSGS: Focal and segmental glomerulosclerosis
GSI: Glomerular sclerosis index
HE: Haematoxylin and eosin
*P. linteus*: *Phellinus linteus*
PLD-HD: High-dose group of *P. linteus* decoction
PLD-LD: Low-dose group of *P. linteus* decoction
PLD-MD: Medium-dose group of *P. linteus* decoction
Scr: Serum creatinine; BUN: urea nitrogen
SD: Podocyte slit diaphrag
TC: Cholesterol
TG: Triglycerides
